# The Viral KSHV Chemokine vMIP-II Inhibits the Migration of Naive and Activated Human NK Cells by Antagonizing Two Distinct Chemokine Receptors

**DOI:** 10.1371/journal.ppat.1003568

**Published:** 2013-08-15

**Authors:** Rachel Yamin, Noa S. Kaynan, Ariella Glasner, Alon Vitenshtein, Pinchas Tsukerman, Yoav Bauman, Yael Ophir, Shlomo Elias, Yotam Bar-On, Chamutal Gur, Ofer Mandelboim

**Affiliations:** The Lautenberg Center for General and Tumor Immunology, The BioMedical Research Institute Israel Canada of the Faculty of Medicine (IMRIC), The Hebrew University Hadassah Medical School, Jerusalem, Israel; University of California-Berkeley, United States of America

## Abstract

Natural killer (NK) cells are innate immune cells able to rapidly kill virus-infected and tumor cells. Two NK cell populations are found in the blood; the majority (90%) expresses the CD16 receptor and also express the CD56 protein in intermediate levels (CD56^Dim^ CD16^Pos^) while the remaining 10% are CD16 negative and express CD56 in high levels (CD56^Bright^ CD16^Neg^). NK cells also reside in some tissues and traffic to various infected organs through the usage of different chemokines and chemokine receptors. Kaposi's sarcoma-associated herpesvirus (KSHV) is a human virus that has developed numerous sophisticated and versatile strategies to escape the attack of immune cells such as NK cells. Here, we investigate whether the KSHV derived cytokine (vIL-6) and chemokines (vMIP-I, vMIP-II, vMIP-III) affect NK cell activity. Using transwell migration assays, KSHV infected cells, as well as fusion and recombinant proteins, we show that out of the four cytokine/chemokines encoded by KSHV, vMIP-II is the only one that binds to the majority of NK cells, affecting their migration. We demonstrate that vMIP-II binds to two different receptors, CX3CR1 and CCR5, expressed by naïve CD56^Dim^ CD16^Pos^ NK cells and activated NK cells, respectively. Furthermore, we show that the binding of vMIP-II to CX3CR1 and CCR5 blocks the binding of the natural ligands of these receptors, Fractalkine (Fck) and RANTES, respectively. Finally, we show that vMIP-II inhibits the migration of naïve and activated NK cells towards Fck and RANTES. Thus, we present here a novel mechanism in which KSHV uses a unique protein that antagonizes the activity of two distinct chemokine receptors to inhibit the migration of naïve and activated NK cells.

## Author Summary

NK cells belong to the innate immune system, able to rapidly kill tumors and various pathogens. They reside in the blood and in various tissues and traffic to different infected organs through the usage of different chemokines and chemokine receptors. KSHV is a master of immune evasion, and around a quarter of the KSHV encoded genes are dedicated to interfere with immune cell recognition. Here, we investigate the role played by the KSHV derived cytokine and chemokines (vIL-6, vMIP-I, vMIP-II, vMIP-III) in modulating NK cell activity. We show that vMIP-II binds and inhibits the activity of two different receptors, CX3CR1 and CCR5, expressed by naïve NK cells and by activated NK cells, respectively. Thus, we demonstrate here a novel mechanism in which KSHV uses a unique protein that antagonizes the activity of two distinct chemokine receptors to inhibit the migration of naïve and activated NK cells.

## Introduction

NK cells are innate immune lymphocytes that comprise approximately 10% of peripheral blood lymphocytes and are phenotypically characterized by the presence of CD56, the expression of NKp46, and the lack of CD3 expression [1]. The majority (approximately 90%) of naïve human NK cells in the peripheral blood express CD56 at intermediate levels (CD56^Dim^) and express high levels of FcγRIII (CD16), whereas a minor population of naive NK cells (approximately 10%) expresses CD56 at high levels and do not express CD16 (CD56^Bright^ CD16^Neg^) [1], [2]. Although mature NK cells predominantly circulate in the peripheral blood, they also reside in several lymphoid and non-lymphoid organs, such as the spleen, tonsils, lymph nodes, liver, lungs, intestine, and the uterus [3]. In most of these organs the predominant NK cell population is CD56^Bright^ CD16^Neg^ [2], [4]. NK cells mediate two major functions: recognition and killing of tumor and virus-infected cells, performed primarily by the CD56^Dim^ CD16^Pos^ subset, and production of immuneregulatory cytokines mainly by the CD56^Bright^ CD16^Neg^ subset [5]. This is also reflected by the receptor repertoire expressed by the CD56^Dim^ CD16^Pos^ and CD56^Bright^ CD16^Neg^ NK cells, as the two subsets express a distinct set of inhibitory and activating receptors and display diversity in their adhesion molecules and chemokine receptors profile [1]–[6].

NK cells express several receptors for CC, CXC, C, and CX3C chemokines, with great heterogeneity in the chemokine receptor repertoire among different NK cell populations, among different individuals and between resting versus activated NK cells. Naïve CD56^Dim^ CD16^Pos^ NK cells express high levels of CXCR1 (IL-8 receptor) and CX3CR1 (Fractalkine receptor) and low levels of CXCR2 and CXCR3 [7], [8]. This NK subset expresses no detectable levels of CC chemokine receptors on their cell surface [9]–[11]. In contrast, CD56^Bright^ CD16^Neg^ NK cells express high levels of CXCR3, CCR5 and CCR7, low levels of CX3CR1, and are negative for CXCR1, CXCR2 and CXCR5 [12]. The differences in chemokine receptor expression correlate with differences in the migratory behavior. The CD56^Dim^ CD16^Pos^ NK cells migrate vigorously in response to Fractalkine (CXC3L1), SDF-1α (CXCL12) and IL-8 (CXCL8), while the chemotaxis of CD56^Bright^ CD16^Neg^ NK cells is stimulated most potently by CXCL10 and CXCL11 (CXCR3 ligands), CXCL12 (CXCR4 ligand), CCL19 and CCL21 (CCR7 ligands) [7], [8], [12].

The expression of chemokine receptors and the corresponding NK cell chemotactic response is also modulated upon cytokine mediated activation. IL-2 and IL-15 induce a strong downregulation of CX3CR1 [13], [14]. Additionally, a significant decrease of CXCR3 expression and chemotaxis to CXCL10 was reported in human NK cells treated for 6 or 24 hours with IL-2, while longer periods of stimulation has been reported to increase the expression of CCR4, CCR5 and CCR8 [10].

KSHV, also named human herpesvirus-8 (HHV-8), belongs to the gamma herpesvirus subfamily. KSHV is the causative agent of Kaposi's sarcoma (KS) and two other lymphoproliferations diseases: multicentric Castleman's disease and primary effusion lymphoma [15]–[18]. There are several forms of KS, but the most severe form occurs in HIV patients and immune suppressed transplant recipients, highlighting the importance of a functional immune system in the control of KSHV infection.

Like other herpesviruses, infection of KSHV has two distinct phases known as lytic replication and latency [19]. During the latent state only a small number of viral proteins are expressed and the viral genome exists as an episome within the host nucleus, while in the lytic state most of the viral genes are expressed, the genome is replicated and packaged, and viruses can egress from the infected cells [20], [21].

Herpesviruses, including KSHV, use several different mechanisms in order to evade recognition by the host immune system. In fact, around 25% of the KSHV-derived proteins have been shown to regulate different aspects of the immune system of the host [22]. Among these proteins are MIR1 (K3) and MIR2 (K5), which downregulate the surface expression of the major histocompatibility complex class I molecules (MHC-I) to prevent cytotoxic T lymphocytes (CTL) attack [23], [24]. However, this mechanism may result in increased susceptibility to NK cells recognition, since MHC-I serve as a ligand for many inhibitory receptors expressed by NK cells inhibiting their cytotoxicity [25]. Consequently, it is not surprising that KSHV evolved to deal with NK cell attack as well: it was shown that MIR2 inhibits the expression of the adhesion molecule ICAM1, which is essential for NK and CTL killing [26]. Additionally, MIR2 downregulates the NK-activating ligands MICA, MICB, and AICL to avoid recognition of the infected cells by the killer receptors NKG2D and NKp80, respectively [27]. Furthermore, previous work from our lab demonstrated that KSHV uses one of its miRNAs, miR-K12-7, to downregulate MICB expression and thus avoid the NKG2D-mediated killing of target cells [28]. Collectively these examples emphasize the importance of NK cells in controlling KSHV infection.

Viral infections stimulate the production of cytokines and chemokines that have a crucial role in inducing the migration of immune cells to areas of infection, in immune regulation, and in anti-viral defense [29], [30]. In this regard, KSHV encodes three chemokine homologues: viral macrophage inflammatory protein (vMIP)-I (ORF K6), vMIP-II (ORF K4) and vMIP-III (ORF K4.1). In addition, KSHV encodes one homologues cytokine called vIL-6 (ORF K2).

While it has been suggested that the viral KSHV-encoded chemokines are related to cellular CC-chemokines, they show limited sequence similarity to their cellular counterparts (∼40%), and direct orthologs are difficult to discern with confidence [31]. Previous papers published since the discovery of the viral chemokines, primarily in the late 90's, focused mainly on identifying the targeted receptors. vMIP-I and vMIP-III were shown to be specific agonists of host CCR8 and CCR4, respectively, and it was demonstrated that Th2 T cells expressing CCR4 manifested increased migration towards vMIP-III in chemotaxis assays [32]. vMIP-II has been shown to bind the chemokine receptors CCR1, CCR2, CCR3, CCR5, and CXCR4. This binding blocked the calcium mobilization elicited by the relevant human chemokines which bind those receptors [31]. In addition, vMIP-II has been reported to bind to a variety of other receptors as a neutral ligand, therefore having the ability to competitively block the actions of cellular chemokines recognizing these same receptors. These receptors include CCR1, CCR2, CCR10, CXCR4 and CX3CR1 [31], [33], [34].

Contradicting results were obtained regarding whether vMIP-II functions as an agonist or as an antagonist. Using a rat model, it was shown that vMIP-II efficiently inhibits the chemotactic activity of activated leukocytes towards MCP-1, MIP-1β, and RANTES [33]. In contrast, vMIP-II was shown to be a selective attractant for Th2 T cells and acts as an agonist for the chemokine receptor CCR8 selectively expressed by this subset [35]. Finally, it has been reported that vMIP-I and vMIP-II can enhance chemotaxis of a monocytic cell line *in-vitro* [36].

Surprisingly, despite the importance of NK cells in fighting KSHV infection, virtually nothing is known regarding whether the KSHV-derived cytokine and chemokines interact with NK cells, and whether and how they affect NK cell activity. Here we show that KSHV-derived vMIP-II directly binds to two distinct chemokine receptors expressed by naïve and activated NK cells, and that vMIP-II antagonizes the activity of these two receptors to inhibit the migration of naïve and activated NK cells.

## Results

### vMIP-II is the only KSHV chemokine that efficiently binds NK cells

**Figure 1:**
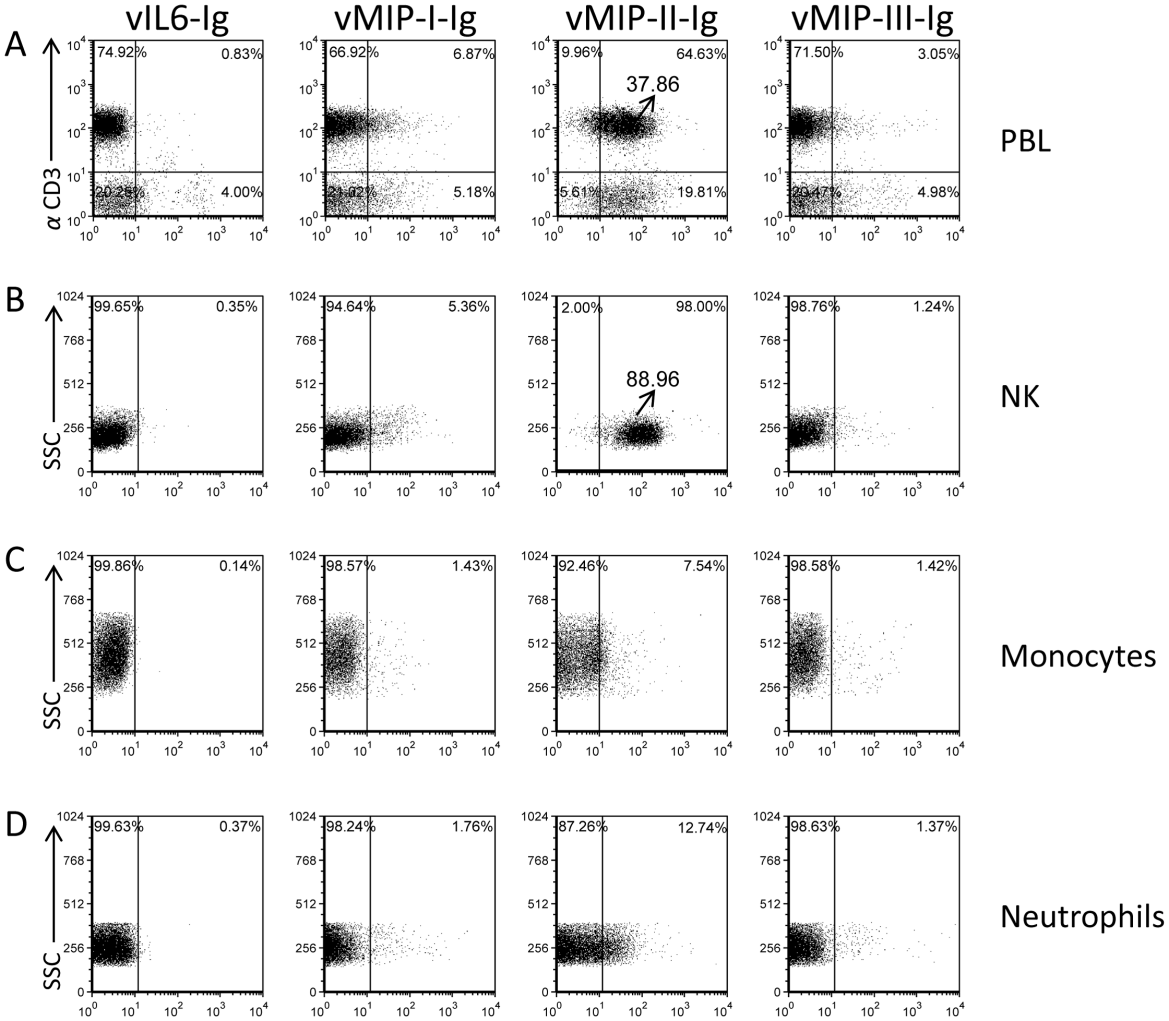
vMIP-II-Ig binds PBLs and naïve NK cells. Freshly isolated PBLs (A), freshly isolated naïve NK cells (B), Monocytes (C) and Neutrophils (D), (the various cell types are indicated in the right of the figure), were stained with different KSHV chemokines fused to human IgG1: vIL6-Ig, vMIP-I-Ig, vMIP-II-Ig or vMIP-III-Ig (X axis, indicated in the top of the figure). PBLs were double stained with the indicated chemokines fused to IgG and with anti-CD3. The percentages of various populations are indicated inside the dot plot. The Median Fluorescence Intensity (MFI) of the vMIP-II-Ig staining of CD3+ cells (A) and of NK cells (B) is indicated and is marked by an arrow. Figure shows one representative staining out of more than 3 performed.

It has been previously suggested that the viral chemokines encoded by KSHV bind a wide spectrum of CC and CXC chemokine receptors [31]. However, it is still practically unknown whether all four KSHV chemokines (including the viral cytokine) bind directly to all immune cell types and how this binding affects immune cell function. To study this issue, we cloned all four KSHV proteins in frame with the human IgG1 Fc domain (which includes a mutation preventing its binding to Fc receptors) and stably expressed them in 293T cells. The proteins were purified on a protein-G column (purity of all fusion proteins used in this manuscript was around 100%, data not shown) and then used to stain peripheral blood lymphocytes (PBLs) (Figure 1A), purified naïve NK cells (Figure 1B), monocytes (Figure 1C) and neutrophils (Figure 1D). As can be seen in figure 1, only a small subset of the PBLs was recognized by vIL-6-Ig, whereas NK cells, neutrophils and monocytes were essentially not recognized. Little binding of vMIP-I-Ig and vMIP-III-Ig was detected to subsets of all immune cells tested, mainly to the lymphocytes and isolated NK cells (Figure 1). Interestingly, most of the lymphocytes and the purified NK cells were recognized by vMIP-II-Ig (Figure 1), while only a subset of monocytes and neutrophils was recognized by this viral chemokine. Thus, of the four KSHV chemokines, vMIP-II was the only one which demonstrated strong binding to the majority of the CD3 positive cells as well as to purified naïve NK cells.

### vMIP-II-Ig mainly recognizes the CD56^Dim^ CD16^Pos^ subpopulation of naïve NK cells, which expresses the CX3CR1 and CXCR1 chemokine receptors

**Figure 2:**
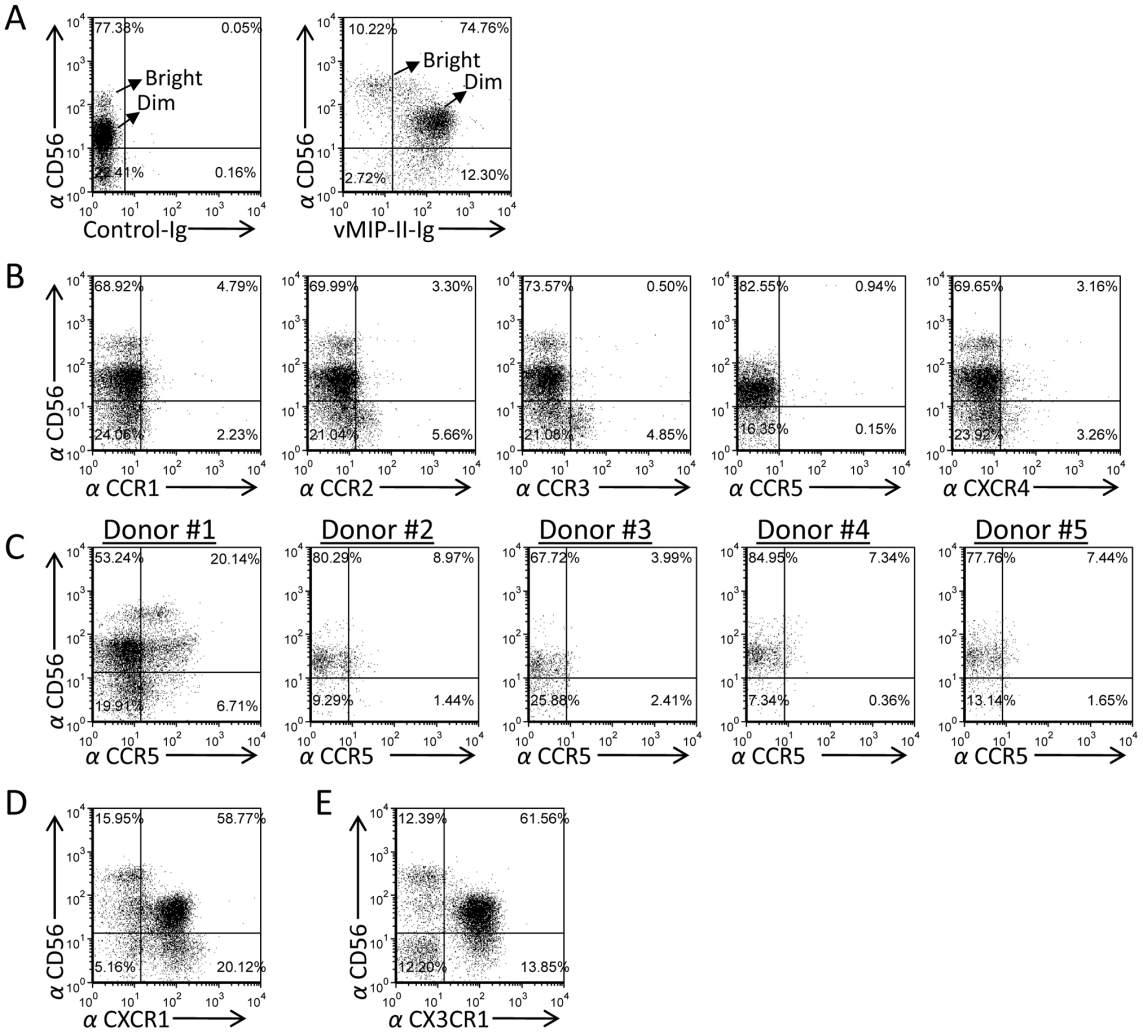
vMIP-II-Ig mainly recognizes the naïve CD56^Dim^ CD16^Pos^ NK cell population which express the CX3CR1 and CXCR1 chemokine receptors. (A) Freshly isolated naïve NK cells were double stained with anti-CD56 mAb together with control-Ig or with vMIP-II-Ig. The CD56^Dim^ CD16^Pos^ and CD56^Bright^ CD16^Neg^ NK cell populations are indicated by an arrow. The percentages of the various populations are indicated. (B) Expression of various chemokine receptors on freshly isolated naïve NK cells. Staining was performed with anti-CD56 mAb together with specific antibodies against CCR1, CCR2, CCR3, CCR5 and CXCR4. The percentages of the various populations are indicated. (C) CCR5 expression varies between different individuals. Staining of various donors (indicated on top of the dot plots) was performed with anti-CD56 mAb together with specific antibodies against CCR5. (D and E) Chemokine receptors expressed primarily by the CD56^Dim^ CD16^Pos^ population. Freshly isolated naïve NK cells were double stained with anti-CD56 mAb together with anti-CXCR1 (D) or anti-CX3CR1 (E). The percentages of the various populations are indicated. Figure shows one representative experiment out of three performed.

The strongest binding of vMIP-II-Ig was observed to purified naïve NK cells (MFI of around 89, Figure 1B). Since it is still unknown whether and how the KSHV-derived chemokines affect NK cell activity, we continued our research concentrating on the vMIP-II interaction with NK cells. To test whether vMIP-II interacts with the two NK cell subsets found in the blood, we performed double staining of freshly isolated naïve NK cells with anti-CD56 and with vMIP-II-Ig. Interestingly, strong vMIP-II-Ig staining was detected mainly in the CD56^Dim^ CD16^Pos^ NK cells sub-population, whereas the CD56^Bright^ CD16^Neg^ NK cells were much less efficiently recognized (Figure 2A, right panel). The binding was specific as no binding was observed when a control fusion protein (Control-Ig, left panel) was used.

It was previously suggested that vMIP-II interacts with several different chemokine receptors including CCR1, CCR2, CCR3, CCR5, and CXCR4 [31]. To test whether the CD56^Dim^ CD16^Pos^ population preferentially expresses these chemokine receptors, we double stained the isolated naïve NK cells with anti CD56 and with anti-CCR1, CCR2, CCR3, CCR5, or CXCR4 mAbs. As can be seen, expression of CCR1, CCR2, CCR3, CCR5 and CXCR4 was hardly detected on the freshly isolated naïve NK cells (Figure 2B). In some donors we observed that a subset of their NK cells express CCR5 (Figure 2C). However, the expression profile of this chemokine receptor was substantially different from the pattern of vMIP-II-Ig staining (compare Figures 2C, donor #1 and Figure 2A, right panel); suggesting that this receptor is not the main one recognized by vMIP-II on naïve NK cells. In contrast, CXCR1 and CX3CR1 demonstrated an expression pattern similar to the vMIP-II-Ig staining (compare Figure 2A to Figures 2D and 2E) and therefore we considered these as possible targets of vMIP-II on naïve NK cells.

### vMIP-II does not induce migration of naïve NK cells

**Figure 3:**
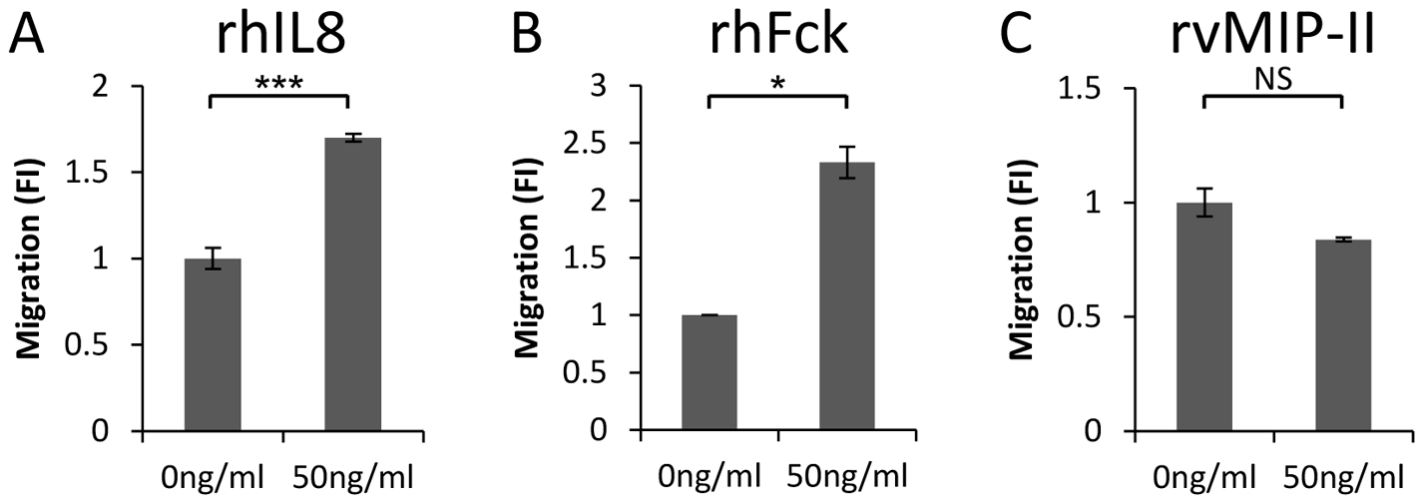
Naïve NK cells migrate towards IL-8 and Fractalkine but not to vMIP-II. Transwell migration assay was performed with the following recombinant proteins: rhIL-8 (A), rhFck (B) or rvMIP-II (C) placed in the bottom chamber and freshly isolated naïve NK cells were placed in the upper chamber. The migration of the NK cells without the appropriate chemokine was set as 1 and the results are presented as fold increase (FI). Rh - recombinant human. Rv - recombinant viral. *P<0.05. ***P<0.001. NS - not significant. Figure shows one representative experiment out of four performed.

Different functions have been suggested for vMIP-II. In some systems it was shown to attract cells, while in other systems it was shown to function as an antagonist [31], [33], [35], [37]. Therefore, we next tested whether naïve NK cells are attracted by the following recombinant chemokines: vMIP-II, IL-8 (a CXCR1 ligand), or Fractalkine (Fck, the CX3CR1 ligand). As can be seen in figure 3, freshly isolated NK cells migrated towards recombinant human (rh) IL-8 and rhFck, while no migration was observed to recombinant viral MIP-II (rvMIP-II). Similar results were obtained with higher concentrations of rvMIP-II or with vMIP-II-Ig (data not shown). Thus, we concluded that vMIP-II might function as an antagonist of NK cells migration.

### vMIP-II does not block the IL-8-mediated migration of naïve NK cells

**Figure 4:**
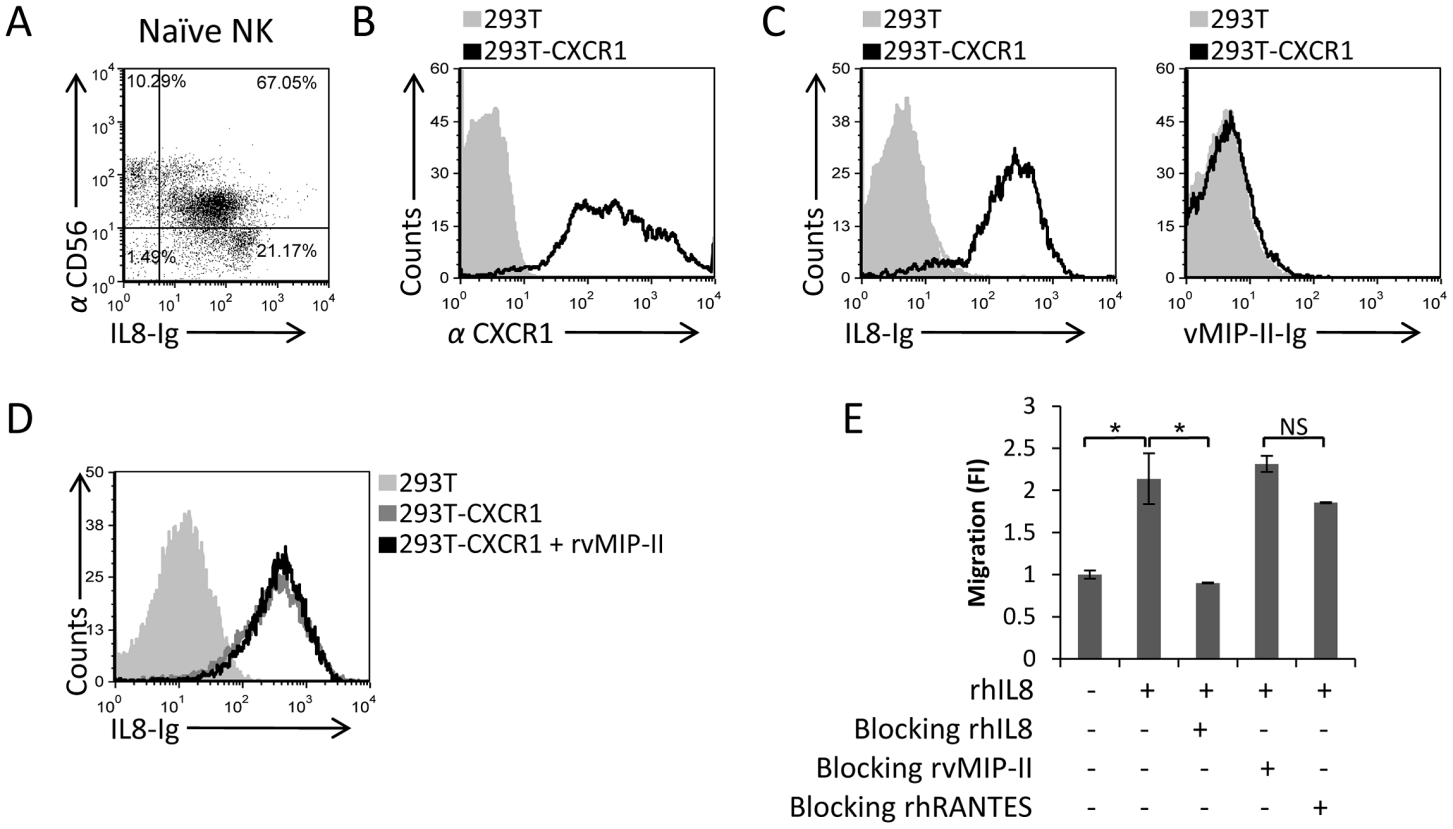
vMIP-II does not block the IL-8-mediated migration. (A) Freshly isolated naïve NK cells were double stained with IL-8-Ig and with anti-CD56. The percentages of the various populations are indicated in the figure. (B) CXCR1 expression on the transfectant 293T-CXCR1 cells (black open histogram) or on the 293T parental cells (filled grey histogram). (C) Binding of IL-8-Ig (left histogram) or vMIP-II-Ig (right histogram) to 293T-CXCR1 transfectant (black open histogram) or to the 293T parental cells (filled grey histogram). (D) 293T-CXCR1 cells were incubated with (black empty histogram) and without (dark gray empty histogram) rvMIP-II for 1 hour in 4°C and then stained with IL-8-Ig. The light gray filled histogram is the staining of IL-8-Ig on the 293T parental cells. (E) Freshly isolated naïve NK cells were incubated at 4°C for 1 hour with and without the proteins indicated in the x axis. RhIL-8 was placed in the bottom chamber and the numbers of migrated cells was determined by FACS following 3 hours incubation at 37°C. The migration of the NK cells without the appropriate chemokine was set as 1 and the results are presented as fold increase (FI). *P<0.05. NS - not significant. Figure shows one representative experiment out of two performed.

We next investigated whether vMIP-II might interact with CXCR1, as this receptor, together with CX3CR1, was expressed predominantly on the entire CD56^Dim^ CD16^Pos^ population (Figure 2D). For this purpose, we initially generated an IL-8-Ig fusion protein (since IL-8 interacts with CXCR1), used it to stain freshly isolated NK cells, and demonstrated that similar to the vMIP-II-Ig staining and in agreement with the expression pattern of CXCR1, the IL-8-Ig fusion protein binds primarily to the naïve CD56^Dim^ CD16^Pos^ and much less to the CD56^Bright^ CD16^Neg^ NK cell population (Figure 4A). Next, we generated transfectants of 293T cells that express the CXCR1 chemokine receptor (Figure 4B) to examine the potential binding of vMIP-II to this receptor. However, we observed that while the CXCR1 transfectants were recognized by IL-8-Ig, vMIP-II-Ig did not bind these cells (Figure 4C, left and right histograms respectively), suggesting that vMIP-II might not interact with CXCR1. Despite this, we cannot rule out that vMIP-II did not interact with CXCR1 as a result of the fusion to the Fc portion. Thus, to further investigate whether vMIP-II interacts with CXCR1, we used the rvMIP-II protein in binding and functional assays. For the binding assay, we incubated the 293T-CXCR1 transfectant cells with rvMIP-II, and then stained the cells with IL-8-Ig. As can be seen in figure 4D, the rvMIP-II protein did not block the binding of the IL-8-Ig to its receptor.

We also tested whether vMIP-II could block the NK cell migration towards IL-8. Naïve NK cells were pre-incubated with and without rhIL-8, rvMIP-II, or rhRANTES (used as a negative control), and then used in migration assays towards IL-8. As can be seen in figure 4E, rhRANTES, which is a ligand for the CCR5, did not block the migration of naïve NK cells towards IL-8, and similarly, rvMIP-II also did not block the NK cells migration. In contrast, the pre-incubation of the naïve NK cells with rhIL-8 abolished their IL-8-mediated migration. Taken together, we concluded that vMIP-II probably does not bind the chemokine receptor CXCR1.

### vMIP-II binds to CX3CR1 and blocks the migration of freshly isolated naïve NK cells towards Fractalkine

**Figure 5:**
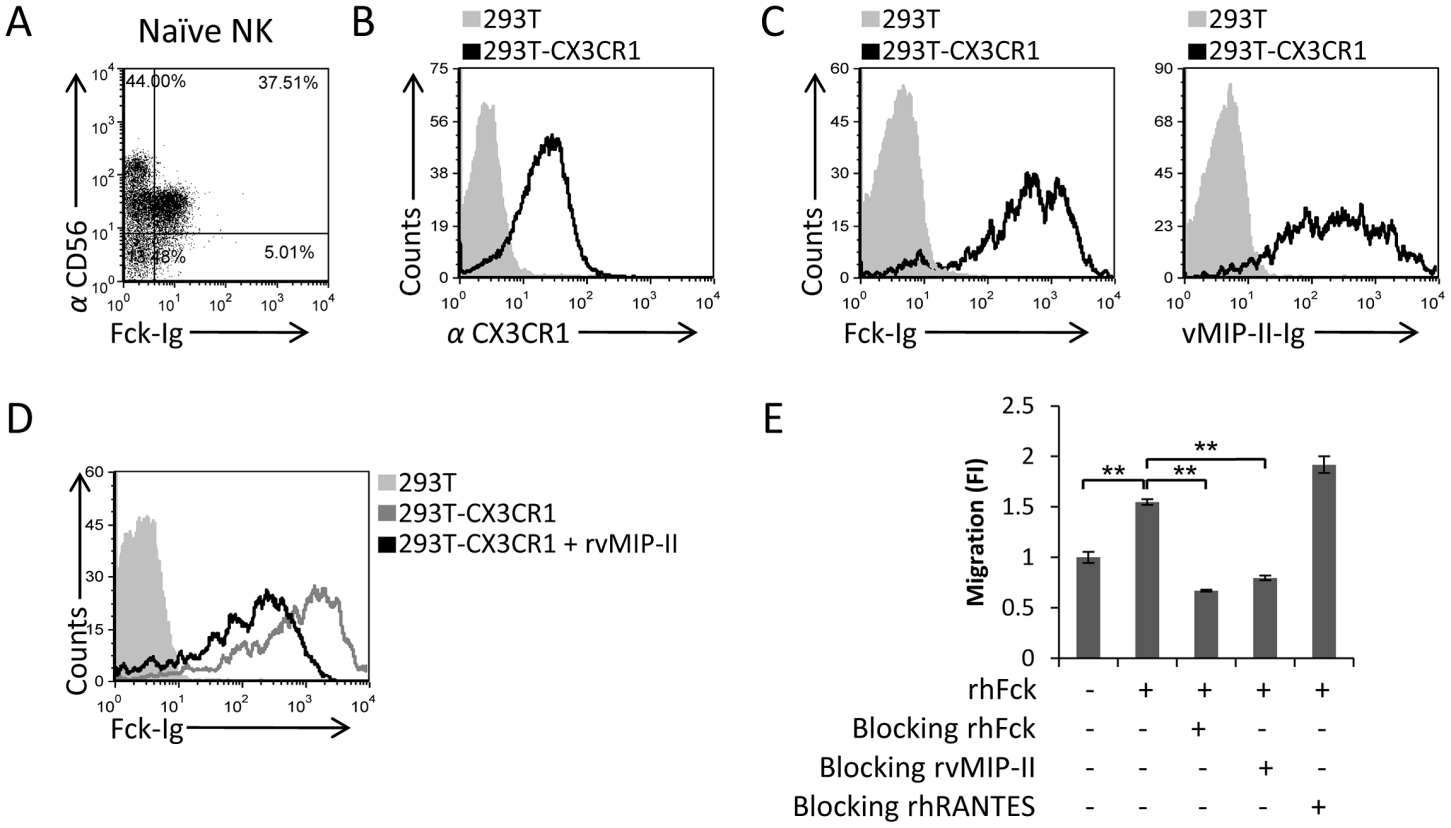
vMIP-II blocks the migration of freshly isolated naïve NK cells to Fractalkine. (A) Freshly isolated naïve NK cells were double stained with Fck-Ig and with anti-CD56 mAb. The percentages of the various populations are indicated in the figure. (B) CX3CR1 expression on the transfectant 293T-CX3CR1 cells (black open histogram) or on 293T parental cells (filled grey histogram). (C) Binding of Fck-Ig (left histogram) or vMIP-II-Ig (right histogram) to 293T-CX3CR1 transfectant (black open histogram) or to the 293T parental cells (filled grey histogram). (D) 293T-CX3CR1 cells were incubated with (black empty histogram) and without (dark gray empty histogram) rvMIP-II for 1 hour in 4°C and then stained with Fck-Ig. The light gray filled histogram is the staining of Fck-Ig on the 293T parental cells. (E) Freshly isolated naïve NK cells were incubated at 4°C for 1 hour with and without the proteins indicated in the x axis. RhFck was placed in the bottom chamber and the numbers of migrated cells was determined by FACS following 3 hours incubation at 37°C. The migration of the NK cells without the appropriate chemokine was set as 1 and the results are presented as fold increase (FI). **P<0.01. NS - not significant. Figure shows one representative experiment out of four performed.

We next tested whether vMIP-II binds the other chemokine receptor that is expressed on the naïve CD56^Dim^ CD16^Pos^ NK cells, CX3CR1. For this we generated another fusion protein composed of the CX3CR1 ligand, Fractalkine, fused to human IgG (Fck-Ig). The fusion protein was produced in 293T cells and purified. In agreement with the expression pattern of its receptor CX3CR1 (Figure 2E), the Fck-Ig protein stained primarily the CD56^Dim^ CD16^Pos^ population (Figure 5A). To test whether vMIP-II can directly interact with CX3CR1, we expressed CX3CR1 in 293T cells (Figure 5B) and tested the binding of Fck-Ig and vMIP-II-Ig to the 293T-CX3CR1 transfected cells. As can be seen, we observed that both Fck-Ig and vMIP-II-Ig bind to the 293T-CX3CR1 transfectant cells (Figure 5C).

To test whether vMIP-II can compete with Fck for binding to its receptor, we pre-incubated the 293T-CX3CR1 cells with rvMIP-II and then stained the 293T-CX3CR1 transfectant cells with Fck-Ig. As can be seen in figure 5D, pre-incubation of the 293T-CX3CR1 cells with rvMIP-II resulted in reduced Fck-Ig staining indicating that both proteins interact with CX3CR1 at similar sites.

Finally, we tested whether vMIP-II affects the Fck-mediated migration of freshly isolated NK cells. Importantly, we observed that pre-incubation of naïve NK cells with vMIP-II completely abolished the Fck-mediated migration of freshly isolated NK cells in a manner similar to that observed with recombinant Fck (rhFck, Figure 5E). Blocking of NK cells migration with the control chemokine rhRANTES had little or no effect (Figure 5E). Blocking of NK cell migration was complete, whereas blocking of Fck-Ig binding to the transfectant cells was partial (Figure 5D), probably because the transfectant cells expresses CX3CR1 at high levels as compared to its expression on naïve NK cells. Thus, we conclude that vMIP-II binds the CX3CR1 chemokine receptor, blocks the binding of Fck to its receptor, and consequently inhibits the migration of naïve NK cells towards Fck.

### vMIP-II binds activated NK cells that express CCR5

**Figure 6:**
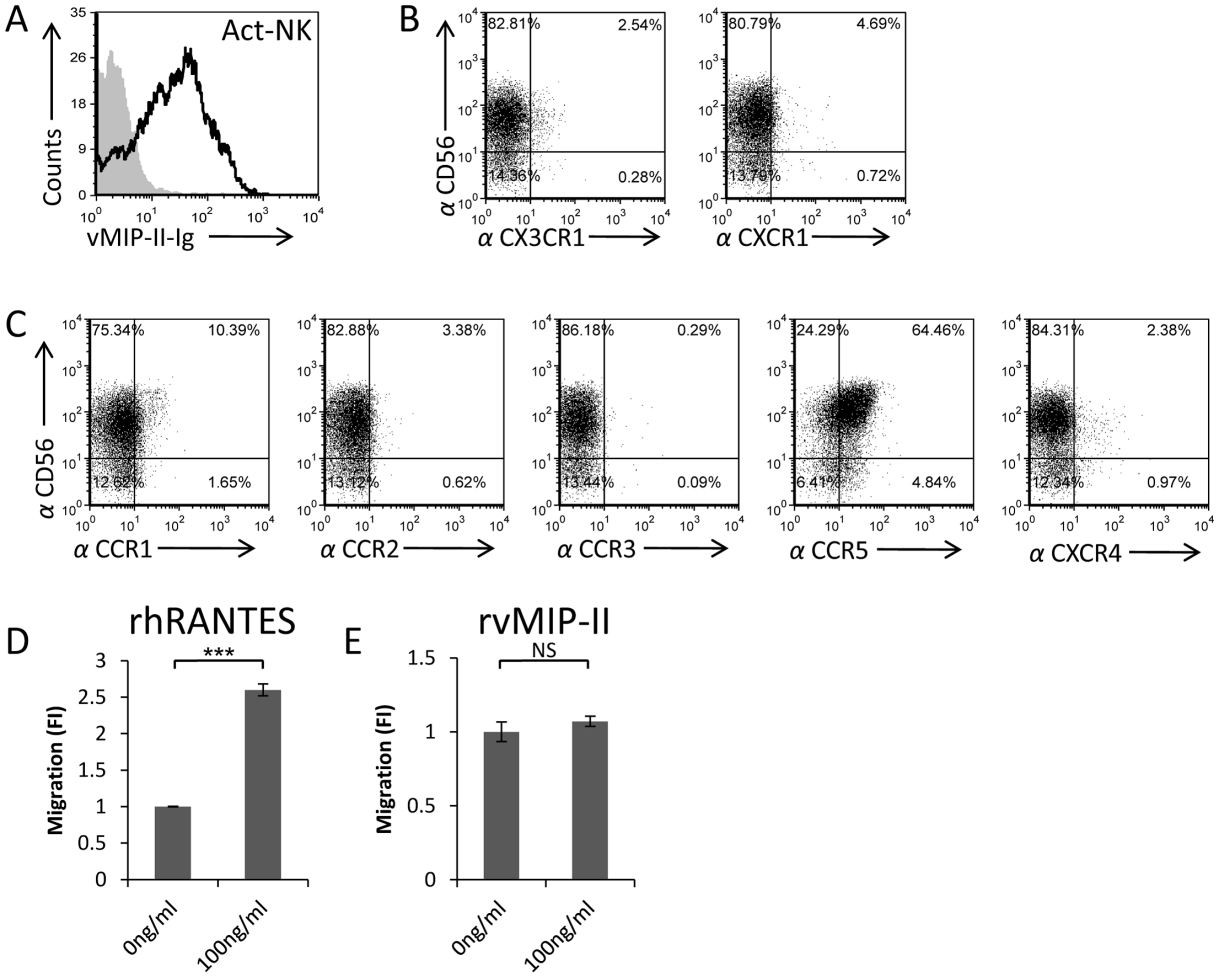
vMIP-II binds activated NK cells that express CCR5. (A) vMIP-II-Ig binding to activated NK cells (Act-NK) (black open histogram). The filled grey histogram is the secondary antibody staining. (B) Activated NK cells were double stained with anti-CD56 together with anti-CX3CR1 (left dot plot) or with anti CXCR1 (right dot plot). Numbers represent percentages. (C) Activated NK cells were double stained with anti-CD56 mAb and antibodies against CCR1, CCR2, CCR3, CCR5 and CXCR4 (indicated in the X axis). Numbers represent percentages. (D and E) rhRANTES (D) or rvMIP-II (E) were placed in the bottom chamber of transwell plates and the migration of activated NK was quantified by FACS following a 3 hours incubation period at 37°C. The migration of the NK cells without the appropriate chemokine was set as 1 and the results are presented as fold increase (FI). ***P<0.001. NS - not significant. Figure shows one representative experiment out of four performed.

It is well known that the expression of killer receptors, chemokine receptors, and the corresponding chemotactic responses of NK cells are modulated upon cytokine stimulation [10], [38], [39]. Therefore, we next tested whether vMIP-II will bind to IL-2-activated NK cells and observed an efficient binding (Figure 6A). Interestingly, binding of vMIP-II to activated NK cells was observed even though the expression of CX3CR1 and CXCR1 almost completely disappeared following activation (Figure 6B). Thus, we concluded that on activated NK cells, vMIP-II interacts with chemokine receptor/s other than CX3CR1. To identify this receptor/s, we performed double staining FACS assays in which we used an anti-CD56 mAb together with anti CCR1, CCR2, CCR3, CCR5, or CXCR4 mAbs (as above, we investigated the expression of these chemokine receptors because they were shown to interact with vMIP-II [31]). As can be seen in figure 6C, of the five receptors tested, CCR5 was the only chemokine receptor that was expressed on the majority of the activated NK cells, demonstrating a staining pattern similar to that of vMIP-II (compare Figures 6A and 6C). Furthermore, as can be seen in figure 6D, RANTES induced activated NK cells chemotaxis in a transwell migration assay, while vMIP-II did not produce any detected NK cells migration (Figure 6E). We demonstrated above that the expression of CCR5 on freshly isolated naïve NK cells was donor specific and that it did not expressed on the entire NK cell population (Figure 2C). In contrast, following activation, CCR5 expression was expressed on the entire NK cell population, irrespectively of whether the NK cells were obtained from donors whose naïve NK cells express CCR5 or not.

Taken together, these results suggested that similar to freshly isolated naïve NK cells, in activated NK cells vMIP-II might also function as an antagonist.

### vMIP-II blocks the chemotaxis of activated NK cells towards RANTES

**Figure 7:**
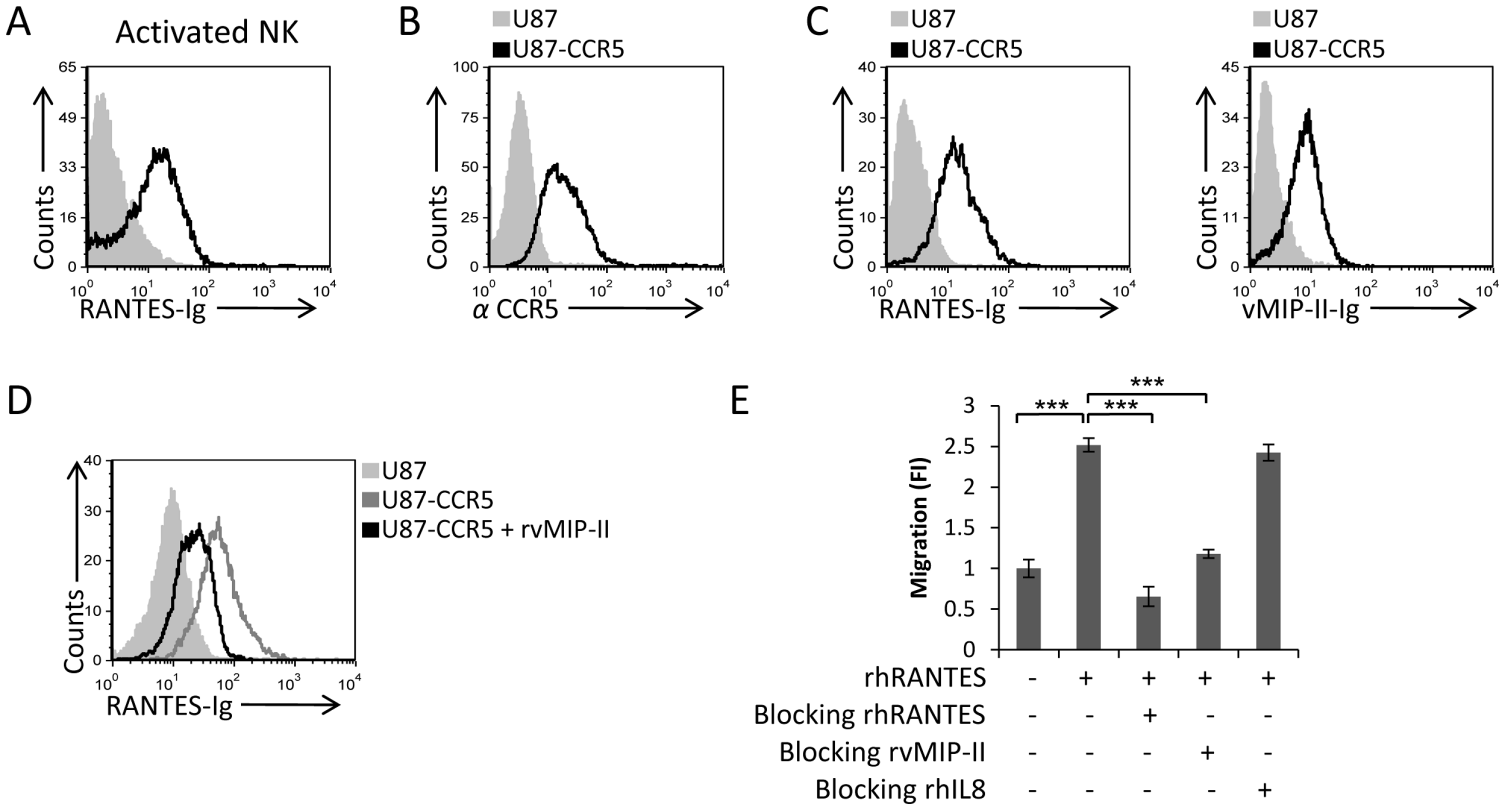
vMIP-II blocks the migration of activated NK cells towards RANTES. (A) Activated NK cells were stained with RANTES-Ig (black open histogram). The filled grey histogram is the secondary antibody staining. (B) U87-CCR5 (black open histogram) or U87 parental cells (filled grey histogram) were stained with anti-CCR5 mAb. (C) FACS staining of RANTES-Ig (left histogram) or vMIP-II-Ig (right histogram) binding to U87-CCR5 cells (staining in both histograms is represented by the black open histogram). The gray filled histograms are the staining of the U87 parental cells with the indicated fusion proteins. (D) U87-CCR5 cells were incubated with (black empty histogram) and without (dark gray empty histogram) rvMIP-II for 1 hour in 4°C and then stained with RANTES-Ig. The light gray filled histogram is the staining of RANTES-Ig on the U87 parental cells. (E) Activated NK cells were incubated at 4°C for 1 hour with and without the proteins indicated in the x axis. RhRANTES was placed in the bottom chamber and the numbers of migrated cells was determined by FACS following 3 hours incubation at 37°C. The migration of the NK cells without the appropriate chemokine was set as 1 and the results are presented as fold increase. ***P<0.001. The figure shows one representative experiment out of four performed.

To examine whether vMIP-II can recognize CCR5, and whether it can block the RANTES binding to CCR5, we initially generated a RANTES-Ig fusion protein and used this protein to demonstrate that RANTES-Ig interacts with IL-2-activated NK cells (Figure 7A). We then used the CCR5 transfected U87 cells (Figure 7B, this cell line was available to us from the NIH AIDS reagent program) to demonstrate that both RANTES-Ig and vMIP-II-Ig bind CCR5 (Figure 7C). Next, we demonstrated that vMIP-II blocks the binding of RANTES to CCR5 as pre-incubation of the CCR5 transfected cells with rvMIP-II significantly reduced the RANTES-Ig binding to the transfectant cells (Figure 7D).

Finally, we demonstrated that vMIP-II not only interferes with the binding of RANTES to CCR5, but also abolished the RANTES mediated migration of activated NK cells. Pre-incubation of the activated NK cells either with rhRANTES or with rvMIP-II completely abolished the migration of activated NK cells towards RANTES, while pre-incubation of the activated NK cells with the control rhIL-8 chemokine had no effect (Figure 7E). The differences between the partial block of binding (Figure 7D) and the complete block of migration (Figure 7E) might be explained, as above, due to the high expression levels of CCR5 on the transfectants.

### KSHV infected cells secrete vMIP-II and inhibit the migration of naïve and activated NK cells

**Figure 8:**
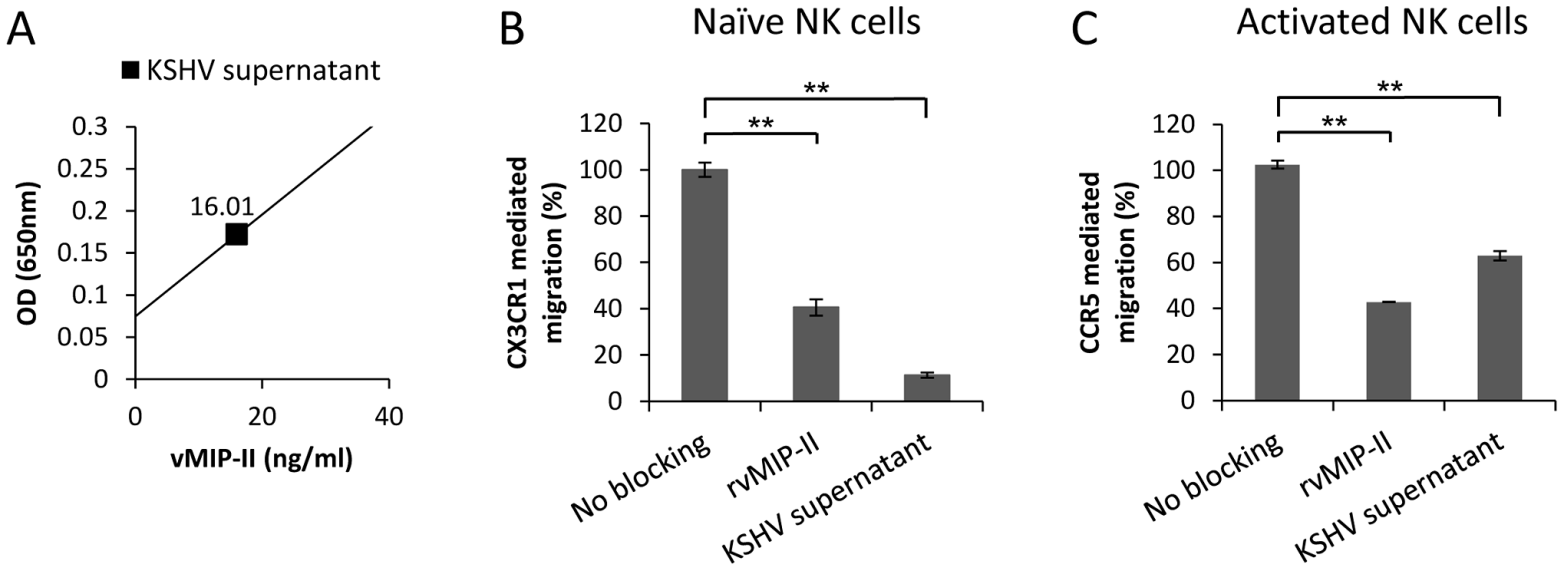
vMIP-II in the supernatant of KSHV infected cells inhibits the migration of naïve and activated NK cells. (A) RvMIP-II was used as standard protein in ELISA assay to determine the concentration of vMIP-II in the supernatant of KSHV infected cells (black square). (B) Naïve NK cells were incubated at 4°C for 1 hour with rvMIP-II or with concentrated supernatant from KSHV infected cells (KSHV supernatant). RhFck was placed in the bottom chamber and the numbers of migrated cells was determined by FACS following 3 hours incubation at 37°C. The migration of the NK cells towards Fck was set as 100%. (C) Activated NK cells were incubated at 4°C for 1 hour with rvMIP-II or with concentrated supernatant from KSHV infected cells (KSHV supernatant). RhRANTES was placed in the bottom chamber and the numbers of migrated cells was determined by FACS following 3 hours incubation at 37°C. The migration of the NK cells towards RANTES was set as 100%. **P<0.01. ***P<0.001. Figure shows one representative experiment out of two performed.

Next we wanted to investigate whether vMIP-II which is secreted following KSHV infection can block NK cell migration. For that we used the iSLK-KSHV cell line that switches from latent state of infection to lytic replication following doxycycline treatment. Initially we determined the concentration of vMIP-II secreted from the KSHV infected cells by using ELISA assay in which the rvMIP-II protein was used as a standard protein and found it to be 16 ng/ml (Figure 8A). Next we pre-incubated naïve or IL-2 activated NK cells together with either rvMIP-II or with the vMIP-II containing supernatants derived from KSHV infected cells and then performed a transwell migration assay towards rhFck (for the naïve NK cells, Figure 8B) or rhRANTES (for the activated NK cells, Figure 8C). The supernatants had to be concentrated 10 fold to achieve efficient inhibition. As can be seen in figure 8B and 8C and similarly to rvMIP-II, the vMIP-II containing supernatants of the KSHV infected cells significantly inhibited the CX3CR1 and CCR5 mediated migration of naïve and activated NK cells, respectively.

## Discussion

In this manuscript, we demonstrate for the first time that the KSHV chemokine vMIP-II antagonizes the activity of two different chemokine receptors expressed by NK cells: CX3CR1 that is expressed on CD56^Dim^ CD16^Pos^ naïve NK cells, and CCR5 that is expressed on activated NK cells. Almost no binding, to any of the cells tested, was observed with vIL6-Ig, with vMIP-I-Ig and with vMIP-III-Ig. Because vMIP-II-Ig did interact with the same tested cells, it is unlikely that the addition of the Ig to vIL6, vMIP-I and to vMIP-II prevented their binding. However, because it is still possible that the lack of binding of vIL6, vMIP-I and vMIP-III resulted from the addition of the Ig these results should be taken with caution. Further investigation which is beyond the scope of this manuscript is needed to resolve this issue.

On freshly isolated naïve NK cells we show that vMIP-II blocks the binding of Fck to CX3CR1 and inhibits CD56^Dim^ CD16^Pos^ NK cells migration. Previous papers showed that vMIP-II and the other viral chemokines bind various chemokine receptors and act as agonists or antagonists depending on the receptor and on the cell type used [31]. Supporting our results, it was shown that vMIP-II inhibits the chemotactic activity of rat activated leukocytes to MCP-1, MIP-1β, RANTES, and Fck [33]. Other studies found that vMIP-II antagonizes the action of MIP-1α, MIP-1β, and RANTES on freshly prepared human monocytes [31], [40].

Fck is a unique molecule that has functional features of both a chemokine (as a secreted form) and an adhesion molecule (as a membrane-bound form) [41]. It was demonstrated that immobilized Fck induces IFN-γ production by NK cells and that soluble Fck significantly enhances NK cell-mediated killing of target cells [8], [42]. However, we did not observe any effect of vMIP-II-Ig on the cytotoxicity or IFN-γ secretion of naïve and activated NK cells (data not shown). It was also shown that NK cells migrate towards Fck in a CX3CR1 dependent manner [8], [43]. As the main cell type permissive for KSHV infection is of endothelial origin, which regularly expresses Fck, it seems reasonable that KSHV developed this vMIP-II-specific mechanism to block NK cell migration to the infection site.

KSHV is not the only virus that evades the immune system by interfering with the CX3CR1-Fck axis. Respiratory syncytial virus (RSV) G protein includes a CX3C motif that was shown to interact with CX3CR1 and competes with Fck. This competition antagonizes the activities of Fck, thereby decreasing the anti-viral response of CX3CR1^Pos^ cells to RSV infection [44]. In the future, it will be interesting to test whether vMIP-II interferes with the RSV G protein binding to CX3CR1 and vice versa.

The CD56^Bright^ CD16^Neg^ NK cells are less cytotoxic; they preferentially release cytokines such as IFN-γ, while CD56^Dim^ CD16^Pos^ NK cells are the principal cytotoxic population. In addition, the CD56^Bright^ CD16^Neg^ subset normally comprises only 10% of the total NK cells in the peripheral blood. Since vMIP-II interacts mainly with the CD56^Dim^ CD16^Pos^ population, it seems as if it is more important for the virus to block NK cell cytotoxicity and not the secretion of cytokines.

We observed here that vMIP-II does not interact with CXCR1. Interestingly, it has been shown that the latency-associated nuclear antigen 1 (LANA-1) protein of KSHV represses IL-8 expression, thus suppressing neutrophils chemotaxis to the infection site [45]. However, there are some reports of several KSHV-encoded genes, expressed during the lytic infection of the virus, that upregulate the expression of IL-8. Among them are vGPCR [46], K1 [47], vFLIP [48], and K15 [49], all together underlining the importance of IL-8 for KSHV infection and pathogenesis. Therefore it is reasonable that the virus will not use its vMIP-II for targeting the CXCR1-IL-8 axis.

Upon cytokine activation, NK cells become more cytotoxic and the expression profile of killer receptors, adhesion molecules, and chemokine receptors changes [13], [14], [50], [51]. We demonstrate here, in agreement with previous studies [13], [52], [53], that following IL-2 activation, the expression of CXCR1 and CX3CR1 is downregulated. This downregulation is concomitant with an upregulation of a different chemokine receptor, CCR5. The changes in the chemokine receptors expression between naïve and activated NK cells could be due to different functions of those receptors in the trafficking of NK cells. It was shown that CX3CR1 is involved mainly in the NK cell exit from bone marrow parenchyma under steady-state [54], while CCR5 regulates the homing of NK cells to inflammatory sites where high concentrations of CCR5 ligands (MIP-1α, MIP-1β, and RANTES) have been found [55].

We demonstrated that vMIP-II binds CCR5 on activated NK cells and inhibits the migration of NK cells towards RANTES. Following KSHV infection, there is a significant upregulation of RANTES, the CCR5 ligand, which is involved in immunoregulatory, inflammatory, and cell proliferation pathways [56], [57]. Therefore, it is important for the virus to target the CCR5-RANTES axis.

Additionally, we aimed to delineate the significance of our findings to native infectious settings. However, KSHV does not have a murine viral analogue and the human KSHV cannot productively replicate in mice [58]. Therefore, we adopted the iSLK-KSHV/DOX inducible *in-vitro* cell based system that is the closest available system to imitate a lytic and latent viral replication environment. We importantly show that KSHV infected cells secrete vMIP-II that efficiently inhibits the migration of both naïve and activated NK cells towards Fck and RANTES, respectively. However to achieve this, the supernatants had to be concentrated 10 times. One must keep in mind that *in-vitro* cell based systems mimic very poorly the physiological concentrations of endogenously secreted components. While cells that comprise the virally infected tissue *in-vivo* are densely packed in a three dimensional essentially solid structure, cells grown in culture are only a very thin two dimensional layer in a great body of liquid media that is many folds the volume of liquid surrounding the cells *in-vivo*. Thus, despite the concentration step, our findings show the importance of vMIP-II in blockade of NK cells during KSHV infection.

Our findings have practical consequences which are not directly related to KSHV. CCR5 is one of the two major co-receptors for HIV-1 entry into the host cells [59]. It has been shown that natural ligands of CCR5 can inhibit HIV-1 infection [60], [61]. As an antagonist of CCR5, vMIP-II could potentially be used therapeutically against HIV. Another possible application for vMIP-II might be in tumor metastasis therapy. It has been shown that RANTES-CCR5 axis plays a key role in the invasiveness of basal breast cancer cells and that CCR5 antagonists (such as maraviroc or vicriviroc, both HIV drugs) blocked tumor invasiveness *in-vitro* and efficiently reduced metastatic colonization *in-vivo* [62]. Thus, vMIP-II can be used in therapy for various cancer types to block the metastasis of the tumor cells and inhibit the aggressiveness of the tumor.

In summary, we demonstrate here a unique mechanism developed by KSHV in which the virus uses a single protein to block NK cells migration at two different stages through the targeting of two different chemokine receptors. Although chemokines are often able to bind with high affinity to more than one receptor, vMIP-II is a unique example, demonstrating the sophistication of the KSHV virus, as it binds with high affinity to more than one subtype of chemokine receptors (CC- and CX3C- chemokine receptors).

## Materials and Methods

### Ethics statement

The NK cells and all other blood subsets that were used in this study were obtained from the blood of healthy volunteers. The institutional Helsinki committee of Hadassah approved the study (Helsinki number 0030-12-HMO). All subjects provided a written informed consent.

### Cells and antibodies

The cell lines used in this paper included 293T and U87 cells. The U87-CCR5 cell line was obtained from the AIDS Research and Reference Reagent Program, Division of AIDS, NIAID, NIH [63]. Monocytes and neutrophils cells isolation was performed as described elsewhere [64]. Monocytes were detected by their physical parameters using a SSC-FSC dot plot, while neutrophils were defined by FACS analysis as CD66b^+^ CD16^+^ cells. PBLs and Naïve NK cells isolation from healthy donors and NK cells activation were performed as previously described [65]. NK cells were defined by being positive for anti-CD56-Phycoerythrin (BD Biosciences) staining and negative for CD3-Allophycocyanin (Biolegend) staining. Staining for the chemokine receptors was performed with conjugated antibodies anti CCR1, anti CCR2, anti CCR3, anti CCR5, anti CXCR4, anti CX3CR1, and anti CXCR1 (all from Biolegend). Alexa Fluor 647 Mouse IgG2b (Biolegend), Alexa Fluor 647 Rat IgG2a (Biolegend), APC Mouse IgG2a (Biolegend), APC Rat IgG2b (Biolegend), APC Mouse IgG2b (Biolegend), and PE Mouse IgG1 (Dako) were used as isotype control antibodies. For secondary antibody staining anti-human APC (Jackson) were used.

### Fusion proteins

Sequences encoding the viral proteins vIL-6 (Gene ID: 4961449), vMIP-I (Gene ID: 4961510), vMIP-II (Gene ID: 4961514) or vMIP-III (Gene ID: 4961436) were amplified by PCR from cDNA isolated from the KSHV positive cell line BCBL1 using the following primers: vIL-6-Ig fwd 5′-CCATGCTAGCGCCGCCACCATGCGCTGGTTCAAGTTGTGG-3′, rev 5′-GGGATCCTTATCGTGGACGTCAGGAGT-3′; vMIP-I-Ig fwd 5′-GGAATTCGCCGCCACCATGGCCCCCGTCCACGTTTTA-3′, rev 5′- GGGATCCGCTATGGCAGGCAGCCGCTG-3′; vMIP-II-Ig fwd 5′- GGAATTCGCCGCCACCATGGACACCAAGGGCATCCTG-3′, rev 5′- GGGATCCCGAGCAGTGACTGGTAATTGC-3′; vMIP-III-Ig fwd 5′- CCATGCTAGCGCCGCCACCATGTGGAGCATGTGCTGGGTG-3′, rev 5′- GGGATCCGGGCATAACCCTTTACCGGC-3′. These PCR-generated fragments were cloned into the mammalian expression vector containing the Fc portion of human IgG1 (mutated to abolish the Fc receptor binding site), generated in 293T cells and Ig-fusion proteins were purified on a protein G column as described [66]. Sequencing of the constructs revealed that cDNA of all Ig-fusion proteins was in frame with the human Fc genomic DNA and were identical to the reported sequences. All Ig-fusion proteins used in this work migrate as a single band on standard non-reduced SDS-PAGE gels and each was regularly assayed by SDS-PAGE to ensure the proteins had not degraded. Protein purity of all Ig fusion proteins used in this study was around 100%.

### Cell transduction

The CX3CR1 and CXCR1 cDNA was amplified from NK cells and HL60 cell line (respectively) and cDNAs were inserted into the pHAGE-DsRED(−)-eGFP(+) lentiviral vector which also contains GFP. 293T cells were co-transfected with the lentiviral vector encoding CXCR1 or CX3CR1, a plasmid encoding the lentiviral Gag/Pol, and a plasmid encoding the VSV-G at a 10∶6.5∶3.5 ratios, respectively. 48 hours after the transfection the supernatant with the viral particles were collected and used to infect 293T cells. The infection percentage was assessed by GFP.

### Migration assay and recombinant proteins

NK cells (0.5×10^6^, 100 µl) were placed in the upper well of a transwell filter (Corning; diameter, 6.5 mm; pore size, 5 µm; 24-well cells clusters). Filters were then plated in bottom wells containing 600 µL migration medium (RPMI 1640 with 1% FCS) supplemented with either rhIL-8 (208-IL-050), rhFck (365-FR-025), rvMIP-II (601-VB-025) or rhRANTES (278-RN-050) (obtained from R&D Systems), as indicated in each figure. At least 3 wells were used for each chemokine. After 3 hours of incubation at 37°C, 5% CO2, the upper chambers were removed and cells in the bottom chamber were collected and counted using a flow cytometer. For the blocking of migration the NK cells were incubated with the indicated recombinant chemokines for 1 hour in 4°C and then loaded into the upper chamber of the transwell. Migration fold increase (FI) was calculated by dividing the number of cells migrating in the presence of chemokines by those migrating toward medium only (control).

### Binding experiments

293T-CXCR1 transfected cells were incubated with rvMIP-II for 1 hour in 4°C and then stained with IL-8-Ig. The 293T-CX3CR1 cells were incubated with rvMIP-II in the same conditions and then stained with Fck-Ig. U87-CCR5 transfectants were stained with RANTES-Ig following 1 hour incubation with rvMIP-II in 4°C.

### KSHV infected cells

iSLK-KSHV cells were kindly obtained from Prof. Rolf Renne. This cell line is doxycycline inducible and can undergo lytic replication after doxycycline treatment. The supernatant from the KSHV infected cells has been collected 72 hours post doxycycline induction and then concentrated by spin filtration using a centricon filter of 3,000 MWCO (Millipore).

### ELISA assay

vMIP-II levels in the supernatant of KSHV infected cells were determined using mouse anti vMIP-II mAb (R&D systems).
